# A UK perspective on the effect of the COVID-19 pandemic on medical student career perceptions

**DOI:** 10.1080/10872981.2020.1810968

**Published:** 2020-08-20

**Authors:** Fayez Elsawy, Rohi Brigid Malik, Mustafah Kazi, Zahra Ladan

**Affiliations:** Faculty of Biology, Medicine and Health, University of Manchester, Manchester, UK

**Keywords:** COVID-19, medical Student, specialty Choice, career Development

## Abstract

The COVID-19 pandemic has altered the normal delivery of medical education globally. Following the cancelation of traditional course components such as elective placements, UK medical students have been able to carry out temporary roles within the NHS. We believe these events may have an effect on medical students’ future choice of specialty.

Dear Editor,

The article by Byrnes et al. inspired us, as medical students in the UK, to consider the effect of COVID-19 on our career perceptions [1]. The point raised regarding the loss of opportunities to explore specialities through student selected research components resonated with our experience. However, we believe that there are other specific aspects of the response to the COVID-19 pandemic in the UK that have influenced our choices regarding our future career.

Byrnes et al. demonstrated that US students’ career perception is largely not affected by the loss of elective placements [1]. However, we feel this is unlikely to be the case in the UK. The current structure of the UK medical school curriculum provides students with a selection of placements to prepare for clinical practice. However, given the breadth of knowledge required, specialty-specific clinical experiences beyond the scope of the curriculum are deprioritised [2]. This contributes to the reliance on electives as an opportunity for students to spend an extended period of time experiencing a specialty of their own interest. Students that have lost this opportunity due to COVD-19 are now disadvantaged in the short term and long term. In the short term, they have missed out on the opportunity to explore a possible career path. Whereas in the long term, these students are competitively disadvantaged when applying to specialty training. As their peers are better able to demonstrate commitment to their specialty through an elective placement [3].

UK Medical students have been able to help the NHS during the COVID-19 pandemic by carrying out roles appropriate to their experience such as working as health care assistants [4]. We believe the recruitment of UK medical students as NHS workers may also influence their future choice of career. The clinical exposure afforded by this opportunity to help has partly mitigated the loss of clinical placements and offered a realistic perspective to working life on the ward in many different specialties. The time spent as temporary NHS workers will have allowed UK medical students to accurately assess factors such as work-life balance and intellectual stimulation offered by their assigned specialty [5]. This assessment is likely to have a bearing on future career choices.

In conclusion, we believe the COVID-19 pandemic resulting in the cancellation of elective placements for medical students and the subsequent opportunity to work as health care assistants within the NHS has affected the career perceptions of UK medical students. The loss of an elective placement will have reduced students’ exposure to their specialty of choice, whereas working in the NHS will have allowed medical students to assess an alternative specialty.
